# HIV, Stigma, and Rates of Infection: A Rumour without Evidence

**DOI:** 10.1371/journal.pmed.0030435

**Published:** 2006-10-31

**Authors:** Daniel D Reidpath, Kit Yee Chan

## Abstract

Although HIV-related stigma is often seen as the greatest barrier to controlling the HIV epidemic, this assertion is still only a hypothesis, say Reidpath and Chan.

The modern concept of a social stigma comes from the work of American sociologist Erving Goffman, who described it as a response to a deeply discrediting attribute that devalues the person [1]. In the medical literature, stigma is almost inevitably written about in terms of adverse social sequelae of a disease—such as leprosy, tuberculosis, epilepsy, schizophrenia, or filariasis [2–6]—or a physical characteristic or functional loss, such as obesity, deafness, or paraplegia [7–9]. The consequences of stigma range from moderate opprobrium at one end of the spectrum to death [10].

## The Role of Stigma in Society

But is stigma always bad for health? Recent research has begun to move beyond the generally descriptive work that has populated the field to consider the possible social and biological functions of stigma [11,12]. This new research has broadened the focus from a singular interest in those who are stigmatised, and the negative effects this has on their lives, to the role of stigma within a population.

When considered at a population level, stigma can be studied as an enduring social process, which inevitably produces negative outcomes for some individuals but might in some circumstances produce positive outcomes for a population. The orchestrated stigmatisation of smoking is a case in point. It appears to reduce the population burden of mortality and morbidity due to tobacco by encouraging some to quit (or never to smoke), although it leaves “recalcitrant” smokers more marginalised by their continued habit [13,14].

## The Stigma of HIV

On any ranked list of stigmatised conditions, HIV would have to lie towards the top. As a global public health issue, HIV remains a huge priority. In 2005, it was estimated that 40.3 million people were living with HIV/AIDS, 4.9 million had newly acquired infections, and 3.1 million had died [15]. The delivery of antiretroviral therapy to everyone infected and the development of new antiretroviral therapies are critical to controlling the epidemic. But equally important is the prevention of new infections.

In 2002, the Joint United Nations Programme on HIV/AIDS (UNAIDS) published a report declaring that the stigma associated with HIV was one of the “greatest barriers” to preventing new infections and alleviating the impact of the disease [16]. In other words, stigma is one of the major determinants of the trajectory of the epidemic. For UNAIDS to make such a declaration, one would expect there to be a considerable body of evidence to back its position. Such a statement naturally suggests that combating the stigma associated with HIV is worthy of substantial economic and human investment.

In the four years since the report was published, the UNAIDS position has remained consistent [17], and is also now well reflected within the World Health Organization (WHO) [18]. The UNAIDS position, however, is complicated. In addition to being a determinant of the global epidemic, UNAIDS also argues that HIV-related stigma is one of the greatest barriers to the provision of treatment, care, and support to people living with HIV/AIDS (PLWHA). A typical description of this relationship is as follows: “Stigma and discrimination both stymie efforts to control the global epidemic and create an ideal climate for further growth. Together, they constitute one of the greatest barriers to preventing further infections, providing adequate care, support and treatment, and alleviating the epidemic's impact” [19].

The two claims are semantically and epidemiologically bound together. The first claim is that stigma is a determinant of the global epidemic. The second is that stigma adversely affects the lives of PLWHA. The second claim is uncontroversial, and is supported by considerable empirical evidence showing that stigma exacerbates the already-heavy burden experienced by PLWHA. Stigma can affect areas of life as diverse as housing, employment, education, and most critically, access to health care [20].

## Where Is the Evidence That Stigma Fuels the Epidemic?

The first claim, linking HIV stigma and the global epidemic, is also treated as a fact—so notorious that it has become the basis for considerable policy and program development [16,21]. The actual evidence base, however, is almost nonexistent. In spite of this lack of evidence, the idea is repeated like a shibboleth [21–31]. With each repetition, its veracity appears to increase.

**Figure 1:**
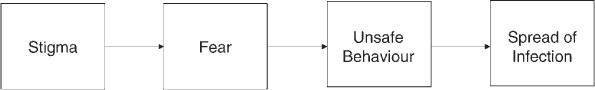
The Argument for the Link between the Stigma of HIV and the Global HIV Epidemic

The argument for the link between HIV stigma and the global epidemic goes something like this: stigma undermines HIV prevention efforts by making a person afraid to engage in safe behaviour or seek testing for fear that these acts would themselves raise suspicion in the minds of others about the person's HIV sero-status [21]. Stigma leads to fear, fear leads to unsafe behaviour, and unsafe behaviour leads to the spread of the infection in the population (Figure 1).

This line of reasoning about the relationship between stigma and the spread of HIV in the population is flawed in two ways. The first flaw is that it ignores the nonlinear dynamics of infectious disease transmission in populations [32–34]. HIV spreads by exploiting a few human behaviours, predominantly sexual intercourse and injection-drug use. Both of these behaviours are associated with a high degree of cultural specificity with respect to who engages in them, who they engage with, and the periods of their lives during which they engage—these are the factors that largely determine the spread of the infection. For this reason, the virus generally takes hold in subpopulations first, such as injection-drug users, commercial sex workers, men who have sex with men, and mobile populations [35,36].

Even if stigma does increase the risk of infection within high-risk groups, it could simultaneously slow the spread of infection from those groups to the general population. Objectionable as it may be to see a lethal infection spread in any part of a population, uncontained spread within a part of the population is better than uncontained spread within the whole population. It is plausible that a social control mechanism, such as stigma, could reduce opportunities for contact between high- and low-risk groups. All other things being equal, under these conditions the spread of the virus across the whole population would be slowed. We are thus suggesting an alternative hypothesis to the UNAIDS position.

The second problem with the claimed relationship between stigma and the spread of HIV is a measurement issue. To establish a causal link between HIV stigma and epidemic progression requires longitudinal data on rates of infection and levels of HIV stigma. Weaker, but nonetheless potentially persuasive, evidence could also be found in an observed correlation between levels of HIV-related stigma and rates of HIV infection across contexts—such as between countries. Currently, no such evidence is available.

Blaming stigma gives too much weight to individual behavioural change as the answer to HIV prevention.

The best supporting evidence comes out of studies such as those recently undertaken in China with mobile populations in which an association was found between increased levels of stigmatising attitudes toward PLWHA and a reported unwillingness to engage in harm-reduction activities [37,38]. Unfortunately, such studies tend to focus only on the spread of infection within particular high-risk subpopulations, and cannot address the question of interaction between populations. Solving the measurement problem requires, among other things, good estimates of the rate of new infections in populations and subpopulations, and a clear understanding of what “HIV stigma” really means.

Data on new HIV infections are sparse. Indeed, UNAIDS itself only reports country estimates of prevalence [15]. Without data on new infections, however, it is hard to establish the correctness of the UNAIDS position. Even where incidence data are available, they often focus on subpopulations rather than whole populations, and this again misses the point about the possible relationship between a social process such as stigmatisation and the spread of HIV in the population [39,40].

It is also difficult to know what exactly is meant, for measurement or intervention purposes, by “HIV stigma” or “HIV-related stigma”. It is not a singular entity. HIV stigma is bound up with pre-existing stigmatising attributes including commercial sex work and injection-drug use [41]. The stigma of one is conflated with the stigma of the others [11]. Separating the effect of pre-existing stigmas from the stigma of the disease alone is important for the development of interventions and for the identification of priorities.

In writing this Essay, our aim is neither to diminish the suffering of PLWHA in the eyes of the reader nor to advocate for the use of HIV stigma as a mechanism to control the spread of the epidemic. Our objective was to draw attention to the lack of evidence supporting the current dominant view on the relationship between stigma and the global spread of HIV. As a driver of policy, the current position closes down potentially important lines of scientific inquiry. Stigma and epidemic control may in fact be two separate problems, but the current position conflates them and halts any consideration of potentially fruitful ways of dealing with them as individual issues.

## Conclusion

Blaming stigma gives too much weight to individual behavioural change as the answer to HIV prevention: stigmatise PLWHA less and engage in harm-reduction behaviours more. It neglects the more-difficult issues relating to the manner in which HIV spreads in populations, the social vulnerabilities it exploits, and the ways in which individuals within subpopulations interact with each other and with members of other subpopulations.

There are some core scientific issues that need to be overcome if the question of the relationship between HIV stigma and the spread of HIV is to be resolved. Whether HIV stigma is one of the greatest barriers to the global control of the epidemic remains a hypothesis. The scientific investigation of it demands significant effort, and should be a matter of priority.

## Abbreviations

PLWHA: people living with HIV/AIDS
UNAIDS: Joint United Nations Programme on HIV/AIDS
WHO: World Health Organization
